# Prostate Cancer Patients' Perceptions Regarding the Relevance of a Digital Rectal Examination During Their Follow‐Up After Radiation Therapy

**DOI:** 10.1002/cam4.70563

**Published:** 2025-01-02

**Authors:** Clément Klein, Ninon Bosc, Sylvie Marty, Laura Calen, Candice Debard, Grégoire Robert, Thibaud Haaser

**Affiliations:** ^1^ Department of Urology University Hospital of Bordeaux Bordeaux France; ^2^ College of Health Sciences University of Bordeaux Bordeaux France; ^3^ Cancer Coordination Centre University Hospital of Bordeaux Bordeaux France; ^4^ Health and Research Ethics Centre University Hospital of Bordeaux Bordeaux France; ^5^ Department of Radiation Oncology University Hospital of Bordeaux Bordeaux France

## Abstract

**Background:**

Prostate cancer is an example of the undervaluation of clinical examinations in care of patients. After external radiotherapy, cancer recurrence is primarily determined biologically by measuring prostate‐specific antigen concentration. Consequently, there is no systematic requirement for the digital rectal examination (DRE). Nevertheless, research has shown that patients attach both practical and symbolic significance to being examined by their physicians. This study aimed to assess how patients perceive DRE omission after prostate cancer radiation therapy.

**Materials and Methods:**

We conducted a survey of 107 men in remission after radiotherapy for prostate cancer in the Radiotherapy Oncology Department of Bordeaux University Hospital, France. The aim of the survey was to assess the significance that patients place on undergoing a DRE as part of their follow‐up care, both from a practical perspective (medical relevance) and a symbolic perspective (influence on the perception of the treating radiation oncologist).

**Results:**

Despite receiving information on the lack of relevance of DRE during follow‐up, 40 of the 100 respondents still perceived a practical benefit of undergoing DRE (pragmatic dimension). On a symbolic level, many patients associated the performance of DRE by their radiotherapy oncologist with impressions of competence, concern for their health and concern for them personally (61%, 63% and 64%, respectively). Although the correlations between the pragmatic and symbolic dimensions were significant, more than one‐third of patients who understood the lack of clinical relevance of DRE still attributed symbolic value to it.

**Conclusions:**

A positive perception of DRE persists among patients, mainly at the symbolic level, including a proportion of patients who understand the low clinical utility of DRE. Importantly, the persistence of these perceptions regarding DRE should not be misconstrued as justification for performing pelvic examinations without clear clinical reasons.

## 1 Introduction

Prostate cancer (PCa) is the second most commonly diagnosed cancer in men, with an estimated 1.4 million diagnoses and 375,000 deaths worldwide in 2020 [1, 2]. Screening for this cancer is performed on an individual–patient basis, following a shared decision‐making process and is not the subject of an organised or mass screening policy. It is based on the combined performance of a prostate‐specific antigen (PSA) assay, with a threshold set at 4 ng/mL, and a digital rectal examination (DRE) in search of a clinical abnormality (palpable nodule or change in consistency of the prostate) [3, 4, 5, 6]. Currently, debates are ongoing regarding the relevance of DRE as a screening modality, with divergent recommendations regarding DRE [3, 4, 5, 6, 7]. However, some studies have indicated that the reason for refusing screening is specifically linked to the performance of DRE, which can lead to a loss of chance [8, 9]. These avoidance behaviours are specifically linked to DRE, especially to its negative representation (fear of pain or discomfort, feeling of shame and attack on masculinity) and are even more marked in certain communities, leading to major inequalities [10, 11, 12].

Exclusive external radiotherapy or external radiotherapy combined with hormonal therapy are standard treatments for prostate cancer according to national and international scientific societies, including the Association Française d'Urologie (AFU), Société Française de Radiothérapie Oncologique (SFRO), American Society for Radiation Oncology (ASTRO), European Society for Radiotherapy and Oncology (ESTRO), European Association of Urology (EAU) and American Urological Association (AUA) [3, 4, 13, 14]. After irradiation of prostate cancer, monitoring methods are based on prostate‐specific antigen (PSA) measurements and clinical examination. The recurrence of prostate cancer after radiotherapy is defined biochemically as an increase in PSA concentration surpassing the absolute nadir threshold of 2 ng/mL (i.e., an increase in PSA concentration of 2 ng/mL above the lowest PSA concentration measured after treatment) [15].

Regarding posttherapeutic follow‐up, the use of DRE to detect recurrence remains controversial. DRE is much less sensitive than PSA measurement, and asymptomatic patients in remission would not benefit from routine DRE because PSA elevation precedes clinical symptoms and local recurrence [16, 17, 18, 19, 20]. Despite agreement among national and international groups regarding conducting PSA measurements at 3 months, every 6 months for 5 years and annually for 10–15 years, these groups differ in whether DRE should be performed during follow‐up. AUA, ASTRO and SFRO do not specifically mention DRE [13, 14]. However, the AFU and ESTRO recommend DRE as a primary examination, with ESTRO noting some reservations regarding its efficacy [3, 4]. Thus, in the Radiation Oncology Department of Bordeaux University Hospital, DRE is not systematically performed during posttreatment follow‐up (unless patients present with significant changes in PSA or clinical symptoms that could benefit from DRE, such as rectal bleeding).

Prostate cancer exemplifies the evolution of medical practices in terms of a potential ‘devaluation’ of clinical examination in favour of technique‐driven approaches based on considerations of performance and perception (this shift is underlined with the increasing emphasis on early utilisation of magnetic resonance imaging (MRI), for example) [21]. However, some studies have shown that patients still attach importance to clinical examination [22, 23]. The potential lack of physical contact between a patient and their healthcare provider represents a significant shift in the traditional doctor–patient relationship. Historically and socially, this relationship has been built on direct interactions between the practitioner and patient's body [24]. These changes in practice must be questioned because of their potential consequences, particularly in terms of unmet patient expectations and impacts on the patient–caregiver relationship [23].

Kravitz and Callahan reported the perceptions of omitted tests, including physical examination, by 56 patients. The omission of tests appeared in two distinct dimensions: a pragmatic dimension (defined as the concrete informative output of the test in the assessment of clinical situations) and a symbolic dimension (which relates to how the test, especially clinical examination, feeds the patient–physician relationship and embeds trust and credibility) [23]. Furthermore, Kadakkia et al. reported a survey of 150 patients with locally advanced, metastatic or relapsed disease treated at the MD Anderson Cancer Center, exploring the pragmatic and symbolic dimensions of physical examination. The results showed that patients valued physical examination for both pragmatic and symbolic dimensions, and the authors concluded that physical examination remains a ‘cornerstone of clinical encounters’ [24].

We conducted a prospective survey to explore patients' perceptions of the omission of DRE and physical examination during follow‐up after radiation therapy for prostate cancer. The main objective of the study was to measure patients' perceptions of the relevance of DRE (pragmatic dimension). The secondary objective was to explore the symbolic dimension of nonperformance of DRE in terms of patients' perception of the care provided by the radiation oncologist. Characteristics such as initial cancer stage, types of treatments or intensity and impact of side effects of treatments were also assessed to determine their potential influence on patients' answers.

## 2 Materials and Methods

### 2.1 Design of the Study and Population

This article reports a prospective single‐centre study based on a questionnaire. The study population consisted of male patients who had been treated with external radiotherapy for prostate cancer at the Radiation Oncology Department of Bordeaux University Hospital. In this department, the current care practice for the follow‐up of patients after radiation therapy does not include a systematic DRE owing to its lack of clinical relevance. Clinical examination and DRE are performed only in cases of significant elevation of PSA, indicating biochemical recurrence (in accordance with the Phoenix criteria) or the emergence of clinical symptoms, especially digestive symptoms, such as rectal bleeding [15]. Patients were informed of the nonsystematic performance of DRE during follow‐up.

All included patients were treated and cared for by the same radiation oncologist (TH), ensuring consistency of clinical practice and information for all patients. The inclusion criteria were as follows: biochemical remission for prostate cancer after radiation therapy (according to the Phoenix criteria) and no indication for DRE or any other clinical examination (as previously described). Patients needing a clinical examination by DRE or others, being currently treated with hormonal therapy or presenting with a biochemical relapse were not included. The study was proposed at the end of the follow‐up consultation for all patients who met the inclusion criteria. After a full information about the aims and the course of the study, and obtaining consent, the included patients completed the questionnaire in a separate room in the absence of the radiation oncologist.

### 2.2 Questionnaire: Creation and Testing

The questionnaire aimed to explore patients' perception of the nonperformance of clinical examination by DRE during their follow‐up after radiotherapy for prostate cancer. The questionnaire was designed by a urological surgeon and a radiation oncologist, and bioethicist by taking up and adapting two articles previously published in the literature. The first article was written by Kravitz et al. and identified through a qualitative study based on semidirective interviews how patients may perceive the value, purpose of function of clinical examination and/or paraclinical tests (such as radiographies or blood analysis) [23]. Patients were included as they hoped that their practitioners would perform a clinical examination or a complementary test. Data collection and content analyses revealed that patients valued clinical and complementary investigations according to two major dimensions. The first is a pragmatic dimension related to everything that the clinical or paraclinical examination can provide concretely (in terms of a preventive and diagnostic approach or the management and monitoring of a pathology). This dimension is linked to patients' expectations in terms of concrete contributions to their health. The second dimension is symbolic and describes the fact that clinical examination or tests play a major role in the interpersonal relationship between a patient and her/his physician. The symbolic dimension is described as a demonstration of a physician's interest in patients' troubles, validation of patients' concerns and demonstration of empathy. Patients also perceived clinical examination or test as a way to establish clinical credibility and to provide specific reassurance. These two dimensions feed patients' evaluations of care. In their study published in 2014, Kadakia et al. reported a questionnaire‐based survey among patients with advanced, metastatic or relapsed cancer undergoing specific oncological treatments at the MD Anderson Cancer Centre, aimed at exploring the perception of the clinical examination [24]. The questionnaire explored the pragmatic and symbolic dimensions as described by Kravitz et al. Of the 26 questions from Kadakia et al. study, 16 questions specifically explored the pragmatic and symbolic dimensions of clinical examination (respectively 9 and 7) and used as a basic framework for the present survey. For our current study, an adaptation was necessary due to some specificities of context: first, the study aimed to include patients in biochemical remission, with no indication for clinical examination; second, the specific clinical examination for localised prostate cancer consists of a DRE (with perceptions and representations reported in the literature); third, the follow‐up of prostate cancer specifically relies on the PSA level measurement with a high sensitivity and specificity regarding which patients are well‐informed. Thus, a total of 10 out of the 26 initial questions were considered relevant and adapted to the specificities of the current study. After translation in French with the help of an English‐native speaker, the survey was submitted to a cognitive pretest to evaluate the intelligibility and relevance of the 10 questions with the partner–patient of the Radiation Oncology Department, 10 patients undergoing radiation therapy for prostate cancer at that time (after information and consent to read and criticise the questions), and six radiation oncologists from the department. During cognitive pretesting, participants were asked to formulate how they understood the questions and to identify whether it was easy to answer them. A final version of the 10 questions was obtained after the collection and integration of all critics from all reviewers.

As presented in Table 1, the five first questions explored the pragmatic dimension, especially patients' perception of the relevance of performing the DRE for monitoring purposes after irradiation (particularly in comparison with performing a PSA assay). The five other questions explored the symbolic dimension of the absence of systematic clinical examination by DRE on the patient's perceptions of care by the radiation oncologist, especially the themes of competence, concern, reassurance and trust. Responses to the 10 questions were based on a Likert scale (strongly agree, agree, undecided, disagree and completely disagree) (Table 1).

**TABLE 1:** Questionnaire.

**Pragmatic dimension of digital rectal examination (DRE)**	
Q.1	I expect to be examined with a DRE during each visit to my radiation oncologist.
Q.2	I am disappointed when my radiation oncologist does not perform a clinical examination with a DRE at each visit.
Q.3	I think the clinical examination with a DRE allows my radiation oncologist to obtain useful information about my cancer.
Q.4	For follow‐up of my cancer, I think that being examined with a DRE by my radiation oncologist is as important as measurement of the PSA level
Q.5	For follow‐up of my cancer, I think that being examined with a DRE by my radiation oncologist is as important as the radiological explorations
**Symbolic dimension of digital rectal examination**	
Q.6	I think the clinical examination with DRE shows that my radiation oncologist is competent.
Q.7	I think the clinical examination with DRE shows that my radiation oncologist cares about my health.
Q.8	I think the clinical examination with DRE shows that my radiation oncologist cares about me personally
Q.9	I trust my radiation oncologist more if he has examined me with DRE.
Q.10	I am reassured if my radiation oncologist examines me with DRE.

### 2.3 Data Collection

Other characteristics of the population were also examined to determine whether they constituted factors influencing the patients' responses (age, time since irradiation, presence of hormonal therapy, duration of hormonal therapy if administered, development of side effects after irradiation, and the impact of these side effects on quality of life according to a numeric scale ranging from 0 to 10).

### 2.4 Statistical Analysis

Data analysis was carried out using the R software environment for statistical computing and graphics (version 4.0.0). The significance level for all statistical tests was set at 0.05, and *p* values were assessed using a two‐sided approach. The patients' sociodemographic information, clinical characteristics and survey responses were summarised using descriptive statistics. Quantitative variables were represented by median and interquartile range, while categorical variables were represented by frequencies. We used Fisher's exact test together with Cramer's *V* test to measure the strength of the relationships between different survey responses, owing to its ability to quantify the association between two variables independently of the sample size. First, we constructed cross‐tabulated tables by crossing the different categories of the variables studied. was then applied to these contingency tables to quantify the strength of the association between the variables, providing Cramer's *V* index. A Cramer's *V* value close to 0 indicated a weak association between variables, whereas a value close to 1 suggested a strong association.

In our analysis, we only evaluated the correlation between Questions 3 and 4 (pragmatic dimension) and questions related to the symbolic dimension of DRE, because Questions 3 and 4 are the most relevant questions in clinical practice.

### 2.5 Ethics

Data were collected, managed and protected in accordance with current French legislation. This study was approved by the Research Ethics Committee of the University Hospital of Bordeaux, France (CER‐BDX 2023–51).

## 3 Results

During the study period (6 September to 15 October 2021), 107 (83%) of 129 patients agreed to complete the questionnaire, with 100 usable questionnaires (i.e., with less than three missing answers). The population characteristics are listed in Table 2. Most patients were aged > 70 years (61%), and 69 had concomitant hormonal therapy combined with radiation therapy. Nearly two‐thirds of patients had been treated more than 1 year ago (63%), and 57% of patients reported side effects due to their treatment. The median impact of side effects on quality of life, rated from 0 to 10, was 5 [0–10].

**TABLE 2:** Population characteristics.

Total	100 (100%)
Age	
< 60 years	4 (4%)
60–65 years	11 (11%)
65–70 years	19 (19%)
70–75 years	23 (23%)
> 75 years	38 (38%)
Missing	5 (5%)
Time since end of treatment	
< 6 months	5 (5%)
6–12 months	18 (18%)
1–2 years	20 (20%)
> 2 years	43 (43%)
Missing	14 (14%)
Concomitant hormonal therapy	
Yes	69 (69%)
No	27 (27%)
Missing	4 (4%)
Duration of hormonal therapy	
< 6 months	17 (24.6%)
6 months – 2 years	22 (31.9%)
> 2 years	24 (34.8%)
Missing	6 (6)
Occurrence of side effects	
Yes	57 (57%)
No	40 (40%)
Missing	3 (3%)
Impact of side effects on quality of life (from 0 to 10)	
Mean (ET)	5.01 (2.74)
Median [IQR]	5.00 [0–10]

**FIGURE 1:**
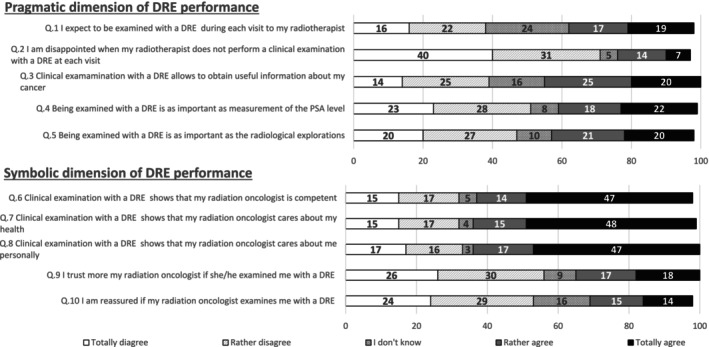
Patient perceptions of DRE: pragmatic and symbolic dimensions (*N* = 100).

With respect to the patients' perception of the relevance of DRE for posttherapeutic monitoring (pragmatic dimension), 40% of patients believed that DRE was as important as PSA assay for posttherapeutic monitoring. Likewise, significant proportions of the study population believed that DRE had informative value for their health state and was as important as imaging examinations (45% and 45%, respectively). A similar proportion expected to be examined at each consultation (36%). Concerning the related symbolic dimension, most patients felt that undergoing clinical examination indicated that their radiation oncologist was competent, cared about their health or even cared about them personally (61%, 63% and 64%, respectively). Some patients identified a link between DRE performance and trust in their physician (35%) or a feeling of reassurance (29%) (Figure 1).

According to the results of Cramer's V test, we found significant correlations between the pragmatic and symbolic dimensions of performing DRE. Specifically, the belief that DRE provides useful information about cancer (Q.3) showed moderate associations with the belief that DRE demonstrates the competence of the radiation oncologist (Q.6, Cramer's *V* = 0.37, *p* < 0.001), that the radiation oncologist cares about the patient (Q.7, Cramer's *V* = 0.35, *p* < 0.001), that the radiation oncologist concerns personally about the patient (Q.8, Cramer's *V* = 0.35, *p* < 0.001) and that trust in the radiation oncologist increases if he performed the examination (Q.9, Cramer's *V* = 0.36, *p* < 0.001).

Similarly, the belief that being examined by the radiation oncologist is as important as measuring the PSA level (Q.4) also showed moderate associations with the same symbolic dimensions: competence (Q.6, Cramer's *V* = 0.35, *p* < 0.001), care (Q.7, Cramer's *V* = 0.31, *p* < 0.001), concerns for the patient personally (Q.8, Cramer's *V* = 0.36, *p* < 0.001), and increased trust (Q.9, Cramer's *V* = 0.41, *p* < 0.001). These results indicate moderate and statistically significant associations between pragmatic beliefs about DRE and symbolic dimensions of the procedure (Table 3).

**TABLE 3:** Correlation between answers concerning the pragmatic and symbolic dimensions of DRE.

Correlation between answers about pragmatic (left) and symbolic (right) dimensions of performing DRE		Contingence index (Cramer's *V*)	*p*
Q.3 Clinical examination with a DRE allows my radiation oncologist to obtain useful information about my cancer.	Q.6 Clinical examination with a DRE shows that my radiation oncologist is competent.	0.37	< 0.001
	Q.7 Clinical examination with a DRE shows that my radiation oncologist cares about my health.	0.35	< 0.001
	Q.8 Clinical examination with a DRE shows that my radiation oncologist cares about me personally.	0.35	< 0.001
	Q.9 I trust my radiation oncologist more if he has examined me with a DRE.	0.36	< 0.001
Q.4 Being examined with a DRE by my radiation oncologist is as important as measurement of the PSA level.	Q.6 Clinical examination with a DRE shows that my radiation oncologist is competent.	0.35	< 0.001
	Q.7 Clinical examination with a DRE shows that my radiation oncologist cares about my health.	0.31	< 0.001
	Q.8 Clinical examination with a DRE shows that my radiation oncologist cares about me personally.	0.36	< 0.001
	Q.9 I trust my radiation oncologist more if he has examined me with a DRE.	0.41	< 0.001

*Note:* Cramer's *V* was used to measure the strength of the relationships between different survey responses. In this table, it indicates the degree of correlation between responses about the pragmatic benefits of DRE and the symbolic dimensions related to the radiation oncologist's perceived competence, care, and trustworthiness. Values close to 0 indicate a weak association, while values closer to 1 suggest a strong association (values are ranging from 0.35 to 0.41 indicating a moderate association). The *p* values show the statistical significance of these correlations, with values less than 0.001 indicating highly significant associations.

When we analysed the data according to whether the patients considered the DRE to be as important as the PSA assay during follow‐up, we found that more than one‐third of the patients who acknowledged the lack of DRE significance still associated DRE performance with competence (19/51 [37%]), concern for their health status (20/51 [39%]) or concern for their well‐being (19/51 [37%]). This pattern persisted when the population was categorised based on their responses to Question 3 (Table 4).

**TABLE 4:** Repartition of answers about symbolic dimension according to pragmatic dimension attributed to DRE.

	Q.4 Being examined by my radiation oncologist is as important as measurement of the PSA level		
	Disagree^a^	Agree^b^	*p*
Q.6 Clinical examination shows that my radiation oncologist is competent			
Agree^b^	19 (37%)	36 (92%)	< 0.001
Disagree^a^	28 (55%)	2 (5%)	
Q.7 Clinical examination shows that my radiation oncologist cares about my health			
Agree^b^	20 (39%)	36 (92%)	
Disagree^a^	28 (55%)	2 (5%)	< 0.001
Q.8 Clinical examination shows that my radiation oncologist cares about me personally			
Agree^b^	19 (37%)	37 (95%)	< 0.001
Disagree^a^	29 (57%)	2 (5%)	
Q,9 I trust my radiation oncologist more if he has examined me			
Agree^b^	4 (8%)	28 (72%)	< 0.001
Disagree^a^	46 (90%)	6 (15%)	

	Q.3 Clinical examination allows my radiation oncologist to obtain useful information about my cancer		
	Disagree^a^	Agree^b^	*p*
Q.6 Clinical examination shows that my radiation oncologist is competent			
Agree^b^	12 (31%)	38 (85%)	< 0.001
Disagree^a^	25 (65%)	3 (7%)	
Q.7 Clinical examination shows that my radiation oncologist cares about my health.			
Agree^b^	12 (31%)	39 (87%)	< 0.001
Disagree^a^	25 (65%)	3 (7%)	
Q.8 Clinical examination shows that my radiation oncologist cares about me personally			
Agree^b^	11 (28%)	41 (91%)	< 0.001
Disagree^a^	26 (67%)	3 (7%)	
Q,9 I trust my radiation oncologist more if he has examined me			
Agree^b^	3 (8%)	27 (60%)	< 0.001
Disagree^a^	36 (92%)	12 (27%)	

^a^ Disagree included totally disagree and rather disagree.

^b^ Agree included totally agree and rather agree.

Finally, regarding the search for influential factors (such as age, duration of hormonotherapy and intensity of side effects), statistical analysis did not find any significant influence on our results (data not shown).

## 4 Discussion

The present study aimed to evaluate the perception of the nonperformance of DRE by patients followed‐up after radiation therapy for prostate cancer, according two complementary dimensions. Although patients were systematically informed by their radiation oncologist that DRE would not be carried out systematically (which corresponds to the current practices of the investigation site), nearly half of the patients still ascribed informative and practical value to this clinical examination. Furthermore, more than half of the patients in this study attached symbolic value to the performance of the DRE, particularly as a sign of competence or personal concern by their radiation oncologist. These results are consistent with other studies highlighting the value that patients attribute to the practice of a clinical examination [24, 25, 26].

It is interesting to note that these positive representations may persist despite the fact that DRE is a penetrating examination performed in a male population. Indeed, literature has reported that DRE constitutes an obstacle to screening for prostate cancer based on negative representations. Studies have specifically explored the perception of DRE conducted for prostate cancer screening and diagnosis, and certain cultural differences have been found [12, 27]. For example, studies among African‐American and Afro‐Caribbean populations showed that these populations were less likely to consent to screening than Caucasian men [10, 11]. These negative perceptions of the DRE are influenced by the unpleasant and embarrassing nature of the examination as well as the perceived attack on masculinity [27, 28]. Screening by pelvic touch thus constitutes a barrier to obtaining consent for prostate cancer screening, with major consequences in terms of disparities and inequalities in access to care [29]. The results of our study contradict these findings in that the performance of DRE seemed to be associated with positive perceptions. However, our study population was very different, comprising men who had prior experience with DRE and who had a cancer diagnosis (in fact, some individuals who declined DRE may have done so to avoid screening due to the fear of a cancer diagnosis) [30]. These important differences in context suggest that our study was oriented around the experience of waiting and not patient apprehension regarding clinical examination. Notably, our findings revealed no patient disappointment regarding the nonperformance of the examination by the radiation oncologist. Studies have revealed unmet patient expectations, a feeling of worry, or even an impression of medical negligence when practitioners do not carry out examinations that the patients consider necessary [23, 24, 25, 26, 27, 28, 29, 30, 31]. The unique nature of DRE in terms of its perception of being somewhat personally invasive and unpleasant by many patients can explain the results of our study [29].

To our knowledge, this is the first study to address patients' perceptions of DRE and the impact of its nonperformance on patients' perception of the radiation oncologist among patients in remission after radiotherapy for prostate cancer. The evolution of care practices in urology involving MRI and PSA measurement provides an interesting context in which to explore the role of clinical examination for the screening or early detection of prostate cancer [32]. Furthermore, the literature consistently emphasises the limited significance and impact of DRE on posttherapeutic care [16, 17, 18, 19, 20].

### 4.1 Limitations

This study has several limitations, such as the small number of included patients, the single‐centre nature of the study and the follow‐up of the patients by a single radiation oncologist. However, this last limitation provides homogeneity to the study population in terms of care and patient information practices. It is also important to consider our results in the light of radiotherapy oncology services. The highly technical nature of these services, lack of physical contact with patients during treatment sessions and distinct treatment pathways in radiotherapy oncology can significantly influence how physicians and patients perceive the importance of clinical examinations [33]. Furthermore, our study was performed after the COVID‐19 pandemic had been ongoing for 18 months, during which some patients were receiving follow‐up via teleconsultation [34]. This prevented any physical or clinical examinations and may have influenced the patients' perceptions of such examinations. Furthermore, if no statistically significant influent factors were found, the socioeconomic status was not collected in this survey, which constitutes another limitation. Finally, the generalisation of our results may be complicated, as the study took place in France with possible cultural specificities and possible limitations due to the process of translation (which was lowered by the help of an English‐native speaker and through cognitive testing).

### 4.2 Ethical Debate

The ethical debate raised by the results of this study is whether radiation oncologists should consider performing a DRE primarily to align with what patients deem necessary for their health, despite any scientific evidence of its clinical relevance. Although our study did not reveal a significant impact on feelings of reassurance or disappointment due to nonperformance of DRE, crucial aspects such as competence and genuine concern for the patient were linked to DRE performance. Exploring and responding to patient preferences in this regard could be valuable in enhancing the therapeutic alliance between patients and caregivers. Some scholars have identified a crucial aspect known as the ‘haptic dimension of care’, which emphasises the sensitive dimension primarily conveyed through personal encounters and touch. Touch holds fundamental value in healthcare and clinical approaches, making it essential for caregivers to acknowledge its necessity in practice [35, 36, 37]. Clinical examination can thus serve an extraclinical function; by leveraging positive perceptions of the clinical gesture, it has the potential to generate beneficial effects, particularly in terms of relational and communication aspects [38, 39]. However, a DRE is an invasive procedure. Contemplating the performance of a medically unnecessary procedure solely to enhance the patient–physician relationship is ethically problematic. Our study was conducted within a broader context of essential ethical reflection, particularly in the ongoing debates about obstetric and gynaecological violence or mistreatment, especially in terms of pelvic examinations [40, 41, 42]. The French National Consultative Ethics Council underlined the ethical imperative of seeking consent and respect for individuals undergoing examinations that affect their privacy [43]. Conducting a pelvic touch examination considered unnecessary to address a need based on inaccurate representations would violate the fundamental principle of respecting individuals' autonomy. Additionally, the literature highlights that while it may serve as a reassuring gesture in establishing the patient–caregiver relationship, clinical examination is also recognised as a tangible method of asserting the physician's authority and dominance over patients [22]. The DRE performance may further accentuate this dynamic. Therefore, it is crucial to enhance the methods of informing individuals and fostering conditions for open discussions regarding these perceptions during follow‐up consultations. This approach is more valuable than adopting a paternalistic stance, aimed at mechanically fulfilling potentially varying and implicit patient requests.

### 4.3 Research Perspectives

This questionnaire‐based study could be complemented by different works. First, similar studies focusing on larger populations (not limited to a population followed by a single doctor) on a national or international scale would allow a complementary evaluation and ensure the measurement of a possible cultural factor. Likewise, as surveys do not permit an in‐depth conceptual analysis of patients' perceptions and demands, qualitative studies based on semidirective interviews could be interesting (close to the study by Kravitz et al.) to validate their hypothesis in the specific context of prostate cancer [23]. Finally, we are also conducting a national survey among French urologists and radiation oncologists aiming to evaluate their concrete care practices towards DRE at screening for prostate cancer and during follow‐up after local treatment.

## 5 Conclusion

Despite systematic information on the lack of scientific relevance of DRE performance during the follow‐up of patients who have undergone radiotherapy for prostate cancer, our study showed a very positive perception of DRE on both the pragmatic and symbolic dimensions. Interestingly, the symbolic dimension remained important, even among some patients who acknowledged the lack of practical importance of the DRE. However, enduring representations that associate clinical examinations with the appreciation of medical competence and meeting the expectations of patients should not be construed as permission to conduct pelvic examinations without genuine clinical justification.

## Author Contributions

**Clément Klein:** conceptualization (equal), data curation (lead), formal analysis (equal), investigation (lead), methodology (lead), validation (equal), writing – review and editing (equal). **Ninon Bosc:** visualization (equal), writing – original draft (equal), writing – review and editing (equal). **Sylvie Marty:** writing – original draft (equal), writing – review and editing (equal). **Laura Calen:** investigation (equal). **Candice Debard:** investigation (equal). **Grégoire Robert:** writing – review and editing (equal). **Thibaud Haaser:** conceptualization (lead), formal analysis (equal), methodology (equal), project administration (lead), supervision (lead), validation (lead), writing – original draft (lead), writing – review and editing (lead).

## Ethics Statement

The authors have nothing to report.

## Consent

According to French regulations, all patients were specifically informed of the study and had the possibility of refusing to fill out the survey (their consent was manifested through filling out the questionnaire).

## Conflicts of Interest

The authors declare no conflicts of interest.

## Data Availability

Data of this article are available on demand.
